# Supplementary material for: Retroperitoneal Lymph Node Dissection in Colorectal Cancer with Lymph Node Metastasis: A Systematic Review
Source: Cancers (Basel). 2023 Jan 10;15(2):455. doi: 10.3390/cancers15020455 (PMC9857277; doi:10.3390/cancers15020455)
Supplement: Supplementary file 1 [file cancers-15-00455-s001.zip › cancers-2135338-supplementary.pdf]

cancers MDPI

Retroperitoneal lymph node dissection in colorectal cancer with lymph node metastasis: a systematic review

Michael G Fadel, Mosab Ahmed, Gianluca Pellino, Shahnawaz Rasheed, Paris Tekkis, David Nicol, Christos Kontovounisios, Erik Mayer

Source	Criteria
**MEDLINE (Ovid)**	(colorectal cancer OR colorectal adenocarcinoma OR CRC OR (colorectal ADJ3 cancer) OR (colorectal ADJ3 adenocarcinoma) OR "colorectal cancer" OR "colorectal adenocarcinoma" OR colorectal tumor* OR colorectal neoplasm* OR (colorectal ADJ3 tumor*) OR (colorectal ADJ3 neoplasm*)).ti,ab<br>(colon cancer OR colon adenocarcinoma OR (colon ADJ3 cancer) OR (colon ADJ3 adenocarcinoma) OR "colon cancer" OR "colon adenocarcinoma" OR colon tumor* OR colon neoplasm* OR (colon ADJ3 tumor*) OR (colon ADJ3 neoplasm*)).ti,ab<br>(rect* cancer OR rect* adenocarcinoma OR (rect* ADJ3 cancer) OR (rect* ADJ3 carcinoma) OR "rectal cancer" OR "rectal adenocarcinoma" OR rect* tumor* OR rect* neoplasm* OR (rect* ADJ3 tumor*) OR (rect* ADJ3 neoplasm*)).ti,ab<br>(bowel cancer OR bowel adenocarcinoma OR (bowel ADJ3 cancer) OR (bowel ADJ3 adenocarcinoma) OR "bowel cancer" OR "bowel adenocarcinoma" OR bowel tumor* OR bowel neoplasm* OR (bowel ADJ3 tumor*) OR (bowel ADJ3 neoplasm*)).ti,ab<br>exp "COLORECTAL NEOPLASMS"/<br>(retroperitoneal lymph node dissection OR "retroperitoneal lymph node dissection" OR RPLND).ti,ab<br>(laparoscopic retroperitoneal lymph node dissection OR "laparoscopic retroperitoneal lymph node dissection" OR L-RPLND).ti,ab<br>"LYMPH NODE EXCISION"/-mt<br>(survival OR surviv* OR patient surviv* OR "patient survival" OR (patient ADJ3 surviv*) OR (cancer ADJ3 surviv*) OR cancer surviv*).ti,ab<br>exp "TREATMENT OUTCOME"/ OR "SURVIVAL RATE"/ 1276468<br>"SURVIVAL ANALYSIS"/ OR "DISEASE-FREE SURVIVAL"/ OR "PROGRESSION-FREE SURVIVAL"/ OR "REMISSION INDUCTION"<br>(recur* OR reoccur* OR cancer recur* OR cancer reoccur* OR recurrent cancer OR "recurrent cancer" OR (cancer ADJ3 recur*) OR (cancer ADJ3 reoccur*) OR restag* OR (cancer ADJ3 restag*)).ti,ab<br>RECURRENCE/ OR "NEOPLASM RECURRENCE, LOCAL
**EMBASE (Ovid)**	(colorectal cancer OR colorectal adenocarcinoma OR CRC OR (colorectal ADJ3 cancer) OR (colorectal ADJ3 adenocarcinoma) OR "colorectal cancer" OR "colorectal adenocarcinoma" OR colorectal tumor* OR colorectal neoplasm* OR (colorectal ADJ3 tumor*) OR (colorectal ADJ3 neoplasm*)).ti,ab<br>(colon cancer OR colon adenocarcinoma OR (colon ADJ3 cancer) OR (colon ADJ3 adenocarcinoma) OR "colon cancer" OR "colon adenocarcinoma" OR colon tumor* OR colon neoplasm* OR (colon ADJ3 tumor*) OR (colon ADJ3 neoplasm*)).ti,ab<br>(rect* cancer OR rect* adenocarcinoma OR (rect* ADJ3 cancer) OR (rect* ADJ3 carcinoma) OR "rectal cancer" OR "rectal adenocarcinoma" OR rect* tumor* OR rect* neoplasm* OR (rect* ADJ3 tumor*) OR (rect* ADJ3 neoplasm*)).ti,ab<br>(bowel cancer OR bowel adenocarcinoma OR (bowel ADJ3 cancer) OR (bowel ADJ3 adenocarcinoma) OR "bowel cancer" OR "bowel adenocarcinoma" OR bowel tumor* OR bowel neoplasm* OR (bowel ADJ3 tumor*) OR (bowel ADJ3 neoplasm*)).ti,ab<br>exp "COLORECTAL CANCER"/ OR "COLORECTAL CARCINOMA"<br>(retroperitoneal lymph node dissection OR "retroperitoneal lymph node dissection" OR RPLND).ti,ab<br>(laparoscopic retroperitoneal lymph node dissection OR "laparoscopic retroperitoneal lymph node dissection" OR L-RPLND).ti,ab<br>*"LYMPH NODE DISSECTION"/<br>(survival OR surviv* OR patient surviv* OR "patient survival" OR (patient ADJ3 surviv*) OR (cancer ADJ3 surviv*) OR cancer surviv*).ti,ab<br>exp "TREATMENT OUTCOME"/ OR "SURVIVAL RATE"/<br>"SURVIVAL ANALYSIS"/

	SURVIVAL/ OR exp "CANCER SURVIVAL"/ OR "DISEASE FREE SURVIVAL"/ OR exp "PROGRESSION FREE SURVIVAL"/ OR exp "RECURRENCE FREE SURVIVAL"/<br>(recur* OR reoccur* OR cancer recur* OR cancer reoccur* OR recurrent cancer OR "recurrent cancer" OR (cancer ADJ3 recur*) OR (cancer ADJ3 reoccur*) OR restag* OR (cancer ADJ3 restag*)).ti,ab<br>"RECURRENT DISEASE"/<br>"CANCER RECURRENCE"/ OR "CANCER REGENERATION"/ OR "CANCER RELAPSE"/
**EMCare (Ovid)**	(colorectal cancer OR colorectal adenocarcinoma OR CRC OR (colorectal ADJ3 cancer) OR (colorectal ADJ3 adenocarcinoma) OR "colorectal cancer" OR "colorectal adenocarcinoma" OR colorectal tumor* OR colorectal neoplasm* OR (colorectal ADJ3 tumor*) OR (colorectal ADJ3 neoplasm*)).ti,ab<br>(colon cancer OR colon adenocarcinoma OR (colon ADJ3 cancer) OR (colon ADJ3 adenocarcinoma) OR "colon cancer" OR "colon adenocarcinoma" OR colon tumor* OR colon neoplasm* OR (colon ADJ3 tumor*) OR (colon ADJ3 neoplasm*)).ti,ab<br>(rect* cancer OR rect* adenocarcinoma OR (rect* ADJ3 cancer) OR (rect* ADJ3 carcinoma) OR "rectal cancer" OR "rectal adenocarcinoma" OR rect* tumor* OR rect* neoplasm* OR (rect* ADJ3 tumor*) OR (rect* ADJ3 neoplasm*)).ti,ab<br>(bowel cancer OR bowel adenocarcinoma OR (bowel ADJ3 cancer) OR (bowel ADJ3 adenocarcinoma) OR "bowel cancer" OR "bowel adenocarcinoma" OR bowel tumor* OR bowel neoplasm* OR (bowel ADJ3 tumor*) OR (bowel ADJ3 neoplasm*)).ti,ab<br>exp "COLORECTAL CANCER"/ OR "COLORECTAL CARCINOMA"/<br>(retroperitoneal lymph node dissection OR "retroperitoneal lymph node dissection" OR RPLND).ti,ab<br>EMCARE (laparoscopic retroperitoneal lymph node dissection OR "laparoscopic retroperitoneal lymph node dissection" OR L-RPLND).ti,ab11<br>*"LYMPH NODE DISSECTION"/<br>(survival OR surviv* OR patient surviv* OR "patient survival" OR (patient ADJ3 surviv*) OR (cancer ADJ3 surviv*) OR cancer surviv*).ti,ab<br>exp "TREATMENT OUTCOME"/ OR "SURVIVAL RATE"/<br>"SURVIVAL ANALYSIS"/<br>SURVIVAL/ OR exp "CANCER SURVIVAL"/ OR "DISEASE FREE SURVIVAL"/ OR exp "PROGRESSION FREE SURVIVAL"/ OR exp "RECURRENCE FREE SURVIVAL"/<br>(recur* OR reoccur* OR cancer recur* OR cancer reoccur* OR recurrent cancer OR "recurrent cancer" OR (cancer ADJ3 recur*) OR (cancer ADJ3 reoccur*) OR restag* OR (cancer ADJ3 restag*)).ti,ab<br>"RECURRENT DISEASE"/<br>"CANCER RECURRENCE"/ OR "CANCER REGENERATION"/ OR "CANCER RELAPSE"/
**CINAHL (EBSCO)**	(colorectal cancer OR colorectal adenocarcinoma OR CRC OR (colorectal ADJ3 cancer) OR (colorectal ADJ3 adenocarcinoma) OR "colorectal cancer" OR "colorectal adenocarcinoma" OR colorectal tumor* OR colorectal neoplasm* OR (colorectal ADJ3 tumor*) OR (colorectal ADJ3 neoplasm*)).ti,ab<br>(colon cancer OR colon adenocarcinoma OR (colon ADJ3 cancer) OR (colon ADJ3 adenocarcinoma) OR "colon cancer" OR "colon adenocarcinoma" OR colon tumor* OR colon neoplasm* OR (colon ADJ3 tumor*) OR (colon ADJ3 neoplasm*)).ti,ab<br>(rect* cancer OR rect* adenocarcinoma OR (rect* ADJ3 cancer) OR (rect* ADJ3 carcinoma) OR "rectal cancer" OR "rectal adenocarcinoma" OR rect* tumor* OR rect* neoplasm* OR (rect* ADJ3 tumor*) OR (rect* ADJ3 neoplasm*)).ti,ab

Table S1. Search strategy for the systematic review on the treatment of retroperitoneal lymph node dissection in colorectal cancer across the four databases (MEDLINE, EMBASE, EMCare and CINAHL), January 1990 – June 2022.

Author, Year	Selection			Comparability			Outcome			Total score
	Representativeness of the exposed cohort	Selection of the non-exposed cohort	Ascertainment of exposure	Outcome not present at start	Comparability of cohorts on the basis of the design or analysis	Study controls for additional factor	Assessment of outcome	Was follow-up long enough	Adequacy of follow up	
Synchronous RPLNM										
**Tentes et al. [26] 2007**										N/A*
**Song et al. [27] 2016**	☆	☆	☆	☆	☆	☆	☆	☆	☆	9
**Ogura et al. [28] 2017**	☆	☆	☆	☆	☆	☆	☆	☆	☆	9
**Bae et al. [29] 2018**	☆	☆	☆	☆	☆	☆	☆			7
**Yamada et al.[30] 2019**	☆		☆	☆	☆	☆	☆	☆	☆	8
**Yamamoto et al. [31] 2019**	☆	☆	☆	☆	☆	☆	☆			7
**Sakamoto et al. [32] 2020**	☆	☆	☆	☆	☆	☆	☆	☆	☆	9
**Lee et al. [33] 2021**	☆	☆	☆	☆	☆	☆	☆	☆	☆	9
Metachronous RPLNM										
**Shibata et al. [17] 2002**	☆		☆	☆	☆	☆	☆	☆	☆	8
**Bowne et al. [34] 2005**	☆		☆	☆	☆	☆	☆	☆	☆	8
**Min et al. [35] 2008**	☆	☆	☆	☆	☆	☆	☆	☆	☆	9
**Dumont et al. [36] 2012**	☆	☆	☆	☆	☆	☆	☆	☆		8
**Razik et al. [37] 2014**	☆		☆	☆	☆	☆	☆	☆	☆	8
**Kim et al. [38] 2020**	☆	☆	☆	☆	☆	☆	☆	☆	☆	9
Synchronous and Metachronous RPLNM										
**Elias et al. [18] 2001**	☆		☆	☆	☆	☆	☆	☆		7
**Choi et al. [10] 2010**	☆	☆	☆	☆	☆	☆	☆	☆	☆	9
**Arimoto et al. [39] 2015**	☆		☆	☆	☆	☆	☆	☆	☆	8
**Gagniere et al. [40] 2015**	☆	☆	☆	☆	☆	☆	☆	☆	☆	9
**Ichikawa et al. [41] 2021**	☆	☆	☆	☆	☆	☆	☆		☆	8

Table S2. Newcastle-Ottawa Scale scoring. * = Newcastle-Ottawa scale not applicable as randomised trial.

Author, Year	Patient selection criteria for performing RPLND
Synchronous RPLNM	
**Tentes et al. [26] 2007**	Randomised Controlled Trial: (1) patients able to tolerate major surgery (no evidence of recent myocardial infarction, cardiomyopathy, or acute pulmonary infection on chest X-ray); (2) tumour distal to splenic flexure and proximal to peritoneal reflection; (3) normal liver function; (4) urea blood level <50 mg/dl; (5) creatinine level <2 mg/dl and (6) performance status >50% (according to Karnofsky performance status scale).
**Song et al. [27] 2016**	(1) Pathological diagnosis of CRC; (2) clinically suspected PALN on the preoperative radiologic studies; (3) suspected PALN metastasis below the renal veins amenable to potentially complete resection.
**Ogura et al. [28] 2017**	MDT discussion of patients with isolated synchronous extra-regional lymph node metastasis situated below the renal veins.
**Bae et al. [29] 2018**	Curative surgery possible and diagnosis of isolated synchronous PALNM with the following exclusion criteria: (1) distant metastases other than to PALNM; (2) lateral pelvic lymph node metastasis and (3) nodal metastasis above the renal vessels.
**Yamada et al.[30] 2019**	(1) Pathological diagnosis of CRC has been made; (2) PALN metastasis is suspected based on preoperative radiologic studies (CT abdomen/pelvis and/or positron emission tomography scan); (3) metastasis is suspected in PALN below the renal vein and (4) the suspected PALN metastasis is amenable to complete resection.
**Yamamoto et al. [31] 2019**	(1) Sufficient medical fitness; (2) suspected PALN metastasis on the preoperative CT abdomen/pelvis (shorter diameter >8 mm, irregular margin or heterogenic contrast pattern) and (3) no signs of disseminated disease, distant metastasis, a widespread primary tumour invading other organs, or upward PALN swelling extending beyond the left renal vein and patients in whom it was determined that curative laparoscopic resection could be performed safely.
**Sakamoto et al. [32] 2020**	MDT discussion: (1) pathological diagnosis of CRC; (2) suspected PALN metastasis on preoperative imaging, such as CT abdomen/pelvis or PET and (3) an assessment that curative resection was possible (i.e. no signs of upward PALN swelling extending above the renal vessels or an obvious invasion of PALN metastases to the great vessels).
**Lee et al. [33] 2021**	Patients with primary CRC with isolated PALNM below the level of the renal vein.
Metachronous RPLNM	
**Shibata et al. [17] 2002**	—
**Bowne et al. [34] 2005**	—
**Min et al. [35] 2008**	—
**Dumont et al. [36] 2012**	MDT Discussion. WHO performance status ≤ 2 with recurrence potentially amenable to curative intent (R0) resection after confirmation of disease control with preoperative chemotherapy, external radiotherapy or both.

**Razik et al. [37] 2014**	Patients with retroperitoneal metastasis with synchronous recurrences which are both amenable to curative resection.
**Kim et al. [38] 2020**	MDT discussion. Single isolated PALN recurrence or multiple PALNs forming a single cluster were considered resectable.
Synchronous and Metachronous RPLNM	
**Elias et al. [18] 2001**	'Highly selected patients': (1) in good general condition; (2) potential for R0 resection; (3) responding to chemotherapy; (4) 'strongly asking for an aggressive approach'.
**Choi et al. [10] 2010**	—
**Arimoto et al. [39] 2015**	Distinctly selected patients who have clinically positive PALN in the very limited local area and who were highly expected to achieve complete resection.
**Gagniere et al. [40] 2015**	MDT discussion. Detailed inclusion criteria not specified by authors.
**Ichikawa et al. [41] 2021**	Patients with pathological PALNs all below the level of the left renal vein and primary CRC.

Table S3. Patient selection factors affecting decision to proceed with retroperitoneal lymph node dissection in colorectal cancer. CRC, colorectal cancer; CT, computed tomography; PALN/M, para-aortic lymph node/metastasis; MDT, multidisciplinary team; PET, positron emission tomography; RPLND/M, retroperitoneal lymph node dissection/metastasis; WHO, World Health Organisation.

Author, Year	Morbidity				
	CD grade 1, n (%)	CD grade 2, n (%)	CD grade 3, n (%)	CD grade 4, n (%)	Total, n (%)
Synchronous RPLNM					
**Tentes et al. [26] 2007**	—	—	—	—	11 (17.7)
**Song et al. [27] 2016**	4 (10.0)		2 (5.0)	0	6 (15.0)
**Ogura et al. [28] 2017**	—	—	—	—	3 (18.8)
**Bae et al. [29] 2018**	—	—	—	—	—
**Yamada et al.[30] 2019**	3 (8.4)	8 (22.2)	3 (8.4)	0	14 (38.9)
**Yamamoto et al. [31] 2019**	—	—	—	—	3 (27.3)
**Sakamoto et al. [32] 2020**	1 (3.4)	5 (17.2)	3 (10.3)	0	9 (31.0)
**Lee et al. [33] 2021**	4 (14.3)	5 (17.9)	1 (3.6)	0	10 (35.7)
Metachronous RPLNM	—	—	—	—	18 (38.3)
**Shibata et al. [17] 2002**					
**Bowne et al. [34] 2005**	0	5 (25.0)	0	0	5 (25.0)
**Min et al. [35] 2008**	—	—	—	—	—
**Dumont et al. [36] 2012**	0	2 (33.3)	0	0	2 (33.3)
**Razik et al. [37] 2014**	—	—	—	—	—
**Kim et al. [38] 2020**	0	18 (37.5)	6 (12.5)	1 (2.1)	25 (52.1)
Synchronous and Metachronous RPLNM	—	—	—	—	—
**Elias et al. [18] 2001**					
**Choi et al. [10] 2010**	0	6	0	0	6 (19.4)
**Arimoto et al. [39] 2015**	2 (11.2)	3 (16.7)	0	0	5 (27.8)
**Gagniere et al. [40] 2015**	—	—	—	—	7 (50.0)
**Ichikawa et al. [41] 2021**	0	0	1 (4.0)	1 (4.0)	2 (8.0)

Table S4. Postoperative morbidity following retroperitoneal lymph node dissection in colorectal cancer. CD, Clavien-Dindo classification [23,24]; RPLNM, retroperitoneal lymph node metastasis.

Author, Year	Re-recurrence, n (%)	Re-recurrence sites, n (%)					
		Liver	Lung	Peritoneum	RPLN	Distant LN	Other
Synchronous RPLNM							
**Tentes et al. [26] 2007**	17 (27.4)	—	—	—	—	—	—
**Song et al. [27] 2016**	9[a] (56.3)	3 (18.8)	2 (12.5)	2 (12.5)	4 (25.0)	2 (12.5)	3 (18.8)
**Ogura et al. [28] 2017**	7 (43.8)	2 (12.5)	0	1 (6.2)	4 (25.0)	2 (12.5)	0
**Bae et al. [29] 2018**	—	—	—	—	—	—	—
**Yamada et al.[30] 2019**	29 (80.6)	—	—	—	—	—	—
**Yamamoto et al. [31] 2019**	4 (36.4)	1 (20.0)	1 (20.0)	0	1 (20.0)	1 (20.0)	0
**Sakamoto et al. [32] 2020**	23 (79.3)	—	—	—	—	—	—
**Lee et al. [33] 2021**	34 (72.3)	—	—	—	—	—	—
Metachronous RPLNM							
**Shibata et al. [17] 2002**	12 (60.0)	—	—	—	—	—	—
**Bowne et al. [34] 2005**	—	—	—	—	—	—	—
**Min et al. [35] 2008**	6 (100)	6 (100)	3 (50.0)	0	0	0	2 (33.3)
**Dumont et al. [36] 2012**	—	—	—	—	—	—	—
**Razik et al. [37] 2014**	21 (48.8)	—	—	—	—	—	—
**Kim et al. [38] 2020**	8 (50.0)	0	2 (12.5)	0	3 (18.8)	2 (12.5)	1 (6.2)
Synchronous and Metachronous RPLNM							
**Elias et al. [18] 2001**	26 (83.8)						
**Choi et al. [10] 2010**	16 (66.7)	4 (16.7)	3 (12.5)	3 (12.5)	7 (29.2)	5 (20.8)	4 (16.7)
**Arimoto et al. [39] 2015**	12 (86.0)	1 (7.1)	6 (43.6)	1 (7.1)	4 (29)		2 (14.2)
**Gagniere et al. [40] 2015**	15 (60.0)	—	—	—	—	—	—
**Ichikawa et al. [41] 2021**	23 (82.1)	4 (14.3)	5 (17.9)	1 (3.6)	11 (39.3)	6 (21.4)	2 (7.1)

Table S5. Sites of re-recurrence following retroperitoneal lymph node dissection in colorectal cancer. a, only includes patients with positive para-aortic lymph nodes on biopsy (n=16); LN, lymph node; RPLNM = retroperitoneal lymph node metastasis.
